# Investigation of the anti-cataractogenic mechanisms of curcumin through in vivo and in vitro studies

**DOI:** 10.1186/s12886-018-0711-8

**Published:** 2018-02-17

**Authors:** Jing Cao, Tao Wang, Meng Wang

**Affiliations:** 1 Department of pharmacy, Linyi People’s hospital of Shandong University, LinYi, 276003 China; 2 Department of Ophthalmology, Linyi People’s hospital of Shandong University, No. 27, Jiefang road, LinYi, Shandong 276003 China

**Keywords:** Curcumin, Cataract, Reactive oxygen, Cell viability, Cell apoptosis

## Abstract

**Background:**

Cataract is the leading cause of blindness in elderly people worldwide, especially in developing countries. Studies to identify strategies that can prevent or retard cataract formation are urgently required. This study aimed to investigate the potential mechanism of the cytoprotective effects of curcumin in in vivo and in vitro experiments.

**Methods:**

Male Wistar rats were randomly divided into three groups: the control group, the model group (administered 20 μmol/kg sodium selenite), and the curcumin group (pretreated with 75 mg/kg body weight curcumin 24 h prior to the administration of sodium selenite). The expression levels of heat shock protein 70 (HSP70), the activities of 8-hydroxy-2-deoxyguanosine (8-OHdG), catalase (CAT), malondialdehyde (MDA), superoxide dismutase (SOD), and glutathione peroxidase (GSH-Px) were assessed by using RT-PCR assay and ELISA. In addition, the cell viability, cell apoptosis, and cell cycle were assessed using a CCK-8 assay and flow cytometry in in vitro studies, followed by RT-PCR analysis to identify the mRNA expression levels of caspase 3, Bcl-2 associated X (Bax), B-cell lymphoma 2 (Bcl-2), cyclooxygenase (Cox-2), c-met, and Slug.

**Results:**

Cataract was successfully established in rats of the model group and the curcumin group through intraperitoneal injection of sodium selenite. The expression levels of HSP70 and the activities of 8-OHdG and MDA in the curcumin group were decreased compared with those in the model group, whereas the activities of CAT, SOD, and GSH-Px were significantly higher than those in the model group (*P* < 0.05). In the in vitro studies, the cell viability and cell apoptosis significantly increased and decreased, respectively, in the curcumin group compared with the model group. Correspondingly, the mRNA expression of caspase-3, Bax, and Cox-2 was lower in the curcumin group than in the model group (*P* < 0.05).

**Conclusions:**

This study suggested that curcumin attenuated selenite-induced cataract through the reduction of the intracellular production of reactive oxygen species and the protection of cells from oxidative damage.

## Background

Cataract is the leading cause of blindness in elderly people worldwide, especially in developing countries [1]. It is estimated that blindness owing to age-related cataracts occurs in approximately 20 million people. At present, the gold standard for the treatment of cataract is the surgical replacement of the cloudy lens with an artificial lens when the cataracts cause problems in daily life [2]. However, the surgery is not an easily accessible treatment option in many countries, especially in low and middle-income countries. Moreover, cataract surgery can cause vision-related complications and risks, such as posterior capsule opacification, especially in infants and children, and place a significant burden on healthcare systems and patients’ quality of life [3–5]. Although the surgical techniques and intraocular lens materials have advanced significantly in the last few decades, the outcomes have not substantially improved. Therefore, studies are urgently required to identify strategies that can prevent or retard cataract formation.

Although the nosogenesis of cataract is not clear, oxidative damage to the eye lens is thought to be an important mechanism in the initiation and progression of cataracts [6]. A series of highly reactive oxygen species (ROS), including superoxide anion (O2^−^), nitric oxide (NO), hydroxyl radicals (OH-), and hydrogen peroxide (H_2_O_2_) has been proven to be implicated in different types of cataract formation [7]. Therefore, considerable efforts have been made to discover effective antioxidative pharmacological agents.

Curcumin, extracted from the rhizome of *Curcuma longa* Linn.*,* is a natural polyphenol. It was first used as an antioxidant to prevent cataract formation in 1996 [8]. Since then, the study of curcumin as a potential anti-cataract agent has been one of the central areas of anti-cataract research [9–12]. Although curcumin has been studied for many years, evidenced-based research is still needed to clarify the biochemical roles in the prevention of cataract formation.

This study aimed to investigate the mechanisms involved in the potential use of curcumin to prevent cataract in in vivo and in vitro studies.

## Methods

Curcumin and sodium selenite of commercially available analytical grades were purchased from Sigma China (Shanghai, China). The lens epithelial cells (LEC) of the HLEB-3 cell line were purchased from iCell Bioscience Inc. (Shanghai, China) and cultured in DMEM supplemented with 10% foetal bovine serum (FBS) in a humidified incubator maintained at 37 °C with an atmosphere of 5% CO_2_.

### Animals and treatments

Ten-day-old male Wistar rats with an average body weight of (25.4 ± 3.7) g were purchased from Shanghai SLAC Laboratory Animal Co., Ltd. (Shanghai, China). All rats were housed at room temperature of (25 ± 1) °C and subjected to a 12/12 h day/night cycle. The rats in all groups were fed a regular diet (Shanghai SLAC Laboratory Animal Co., Ltd., Shanghai, China) with distilled water ad libitum for a period of 2 weeks. The animal experiments were approved by the ethics committee of LinYi (LW2017003).

The rats were randomly allocated into three groups: the control group (*n* = 6), administered physiological saline; the model group (n = 6), administered sodium selenite (intraperitoneal injection with signal dose of 20 μmol/kg body weight sodium selenite) to induce cataract; and the curcumin group (*n* = 6), administered a curcumin pretreatment before sodium selenite (75 mg/kg body weight curcumin, orally administered 24 h before selenium administration), as described in previous studies [11, 13]. The eyes were examined every other day.

After treatment for 2 weeks, the rats were killed by cervical dislocation under anaesthesia and the lenses were dissected out, washed with ice-cold saline, and frozen at − 70 °C until further use.

### Biochemical examinations

A 10% homogenate was prepared in aqueous buffers of 0.1 M Tris-HCl (pH 7.4) and centrifuged at 10000 rpm at 4 °C for 30 min. The supernatant was isolated and used to test 8-oxo-deoxyguanosine (8-OHdG), malondialdehyde (MDA), catalase (CAT), superoxide dismutase (SOD), and glutathione peroxidase (GSH-Px) by using ELISA kits (Nanjing Jiancheng Biological Engineering Institute, Jiangsu, China) in accordance with the manufacturer’s instructions.

### Intracellular O_2_^−^ concentration detection

The intracellular ROS concentration was detected in accordance with previous studies [14, 15]. Briefly, the cells in each group were stained with 5 μM DHE (dihydroethidium, Invitrogen Shanghai, China) at 37 °C in the dark for 30 min. Afterwards, the cells were examined by using a fluorescence activated cell sorter with excitation at 480–535 nm and emission at 590–610 nm.

### Cell viability analysis

The HLEB-3 cells were seeded at a density of 1 × 10^4^ cells/cm^2^ in a 96-well plate, grown overnight, and then administered treatment as appropriate. The cells were treated with 200 μM H_2_O_2_ (H_2_O_2_ group), 200 μM H_2_O_2_ plus curcumin (0.2 mM in 2% acetonitrile solution, H_2_O_2_ + Curcumin group) for 24 h; cells without any treatment served as the control (Control group). The cell counting kit-8 (CCK-8) solution was then added to each well and incubated at 37 °C for 4 h. At the end of this treatment, absorbance was measured at 450 nm. The cell growth inhibition rate was calculated as described in reference [16]. The assay was repeated in triplicate.

### Cell cycle and cell apoptosis assay

HLEB-3 cells were seeded in 6-well plates and treated with 200 μM H_2_O_2_ (H_2_O_2_ group), 200 μM H_2_O_2_ plus curcumin (0.2 mM in 2% acetonitrile solution, H_2_O_2_ + Curcumin group) for 24 h, and cells without any treatment served as the control. The cells were harvested, washed with phosphate-buffered saline (PBS), and stained with annexin V-FITC/PI (Becton Dickinson, Franklin Lakes, NJ, USA) at room temperature for 15 min. Subsequently, the cells were analysed by using flow cytometry.

For the cell cycle analysis, the three different treatments of HLEB-3 cells were applied for 48 h. Subsequently, the cells were harvested by trypsinisation, washed twice in PBS, and fixed in 70% ethanol at 4 °C overnight. The cells were then washed, resuspended in cold PBS, and treated with staining buffer at 37 °C in the dark for 30 min. The cell cycle was analysed by using flow cytometry.

### Real-time PCR

Total RNA from the lenses or cells was extracted in each group by using Trizol reagent (Sigma). RT-PCR was performed as previously described [17]. The primers for heat shock protein (HSP70), caspase-3, Bcl-2 associated X (Bax), B-cell lymphoma 2 (Bcl-2), cyclooxygenase (Cox-2), c-met, and Slug are displayed in Table 1. The reactions were conducted in triplicate and the results are shown from three independent experiments.

**Table 1:** The sequences of primers for RT-PCR

Primer sequence (5′-3′)	Gene
TTT CTG GCT CTC AGG GTG TT	HSP70-f
CTG TAC ACA GGG TGG CAG TG	HSP70-r
GACTTCGCCGAGATGTCCAGC	Bcl-2-f
CCGAACTCAAAGAAGGCCACAAT	Bcl-2-r
GTGCTATTGTGAGGCGGTTGT	Caspase 3-f
TCCATGTATGATCTTTGGTTC	Caspase 3-r
AGAAGGCTAAAGGAAACGAA	c-Met-f
GGACCGTCAAGAAGTAAATAAA	c-Met-r
CCCTGAGCATCTACGGTTTG	Cox-2-f
CAGTATTAGCCTGCTTGTCT	Cox-2-r
ATTTATGCAATAAGACCTATTCT	Slug-f
AGGCTCACATATTCCTTGTCACA	Slug-r
CTGACGGCAACTTCAACTGGG	Bax-f
GGAGTCTCACCCAACCACCCT	Bax-r

### Enzyme-linked immunosorbent assay (ELISA)

The protein expression levels of MDA, SOD, and CAT in the three treatment groups were measured by using individual ELISA kits in accordance with the manufacturer’s instructions.

### Statistical analysis

All data are expressed as the mean ± standard deviation. Comparisons between two groups were examined by Student’s *t* test, computed by using GraphPad Prism (GraphPad Software, San Diego, CA, USA). A value of *P* < 0.05 was considered to indicate statistical significance.

## Results

### Construction of cataract model

After the injection of sodium selenite for 2 weeks, the lens of rats in the model group took on a white appearance and lost transparency; however, the transparency of rats in the curcumin group was improved compared with those in the model group.

### *Determination of* in vivo *levels of HSP70, 8-OHdG, MDA, CAT, SOD, and GSH-Px*

**Fig. 1:**
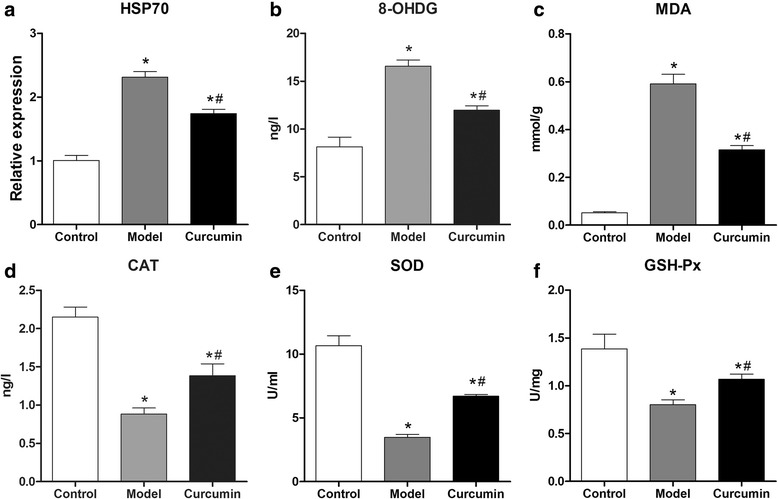
The relative expression level of HSP70 (**a**) and the content of other biochemical index (**b-f**) in the control, model, and curcumin groups in in vivo experiments. * *P* < 0.05 compared with the control group, # *P* < 0.05 compared with the model group

HSP70 levels in the lens were further determined by the RT-PCR analysis of each group. As shown in Fig. 1a, the HSP70 level in the model group was significantly higher than that in control group (*P* < 0.05); however, it was significantly reduced in the curcumin group, which suggested that curcumin could reverse some of the effect of selenite. To further investigate the effect of curcumin, the activities of 8-OHdG, MDA, CAT, SOD, and GSH-Px were measured by using ELISA assays. As shown in Fig. 1b–f, the activities of 8-OHdG and MDA significantly increased in the model group and the curcumin group compared with those in control group (*P* < 0.05). In addition, their expression levels were significantly lower in the curcumin group than those in model group (*P* < 0.05). The activities of CAT, SOD, and GSH-PX, showed an opposite trend in expression: a decrease in the model group and curcumin group was observed compared with those in the control group (*P* < 0.05). Similarly, their activities were higher in the curcumin group than in the model group (*P* < 0.05).

### Evaluation of intracellular superoxide (O_2_^−^) level by DHE

**Fig. 2:**
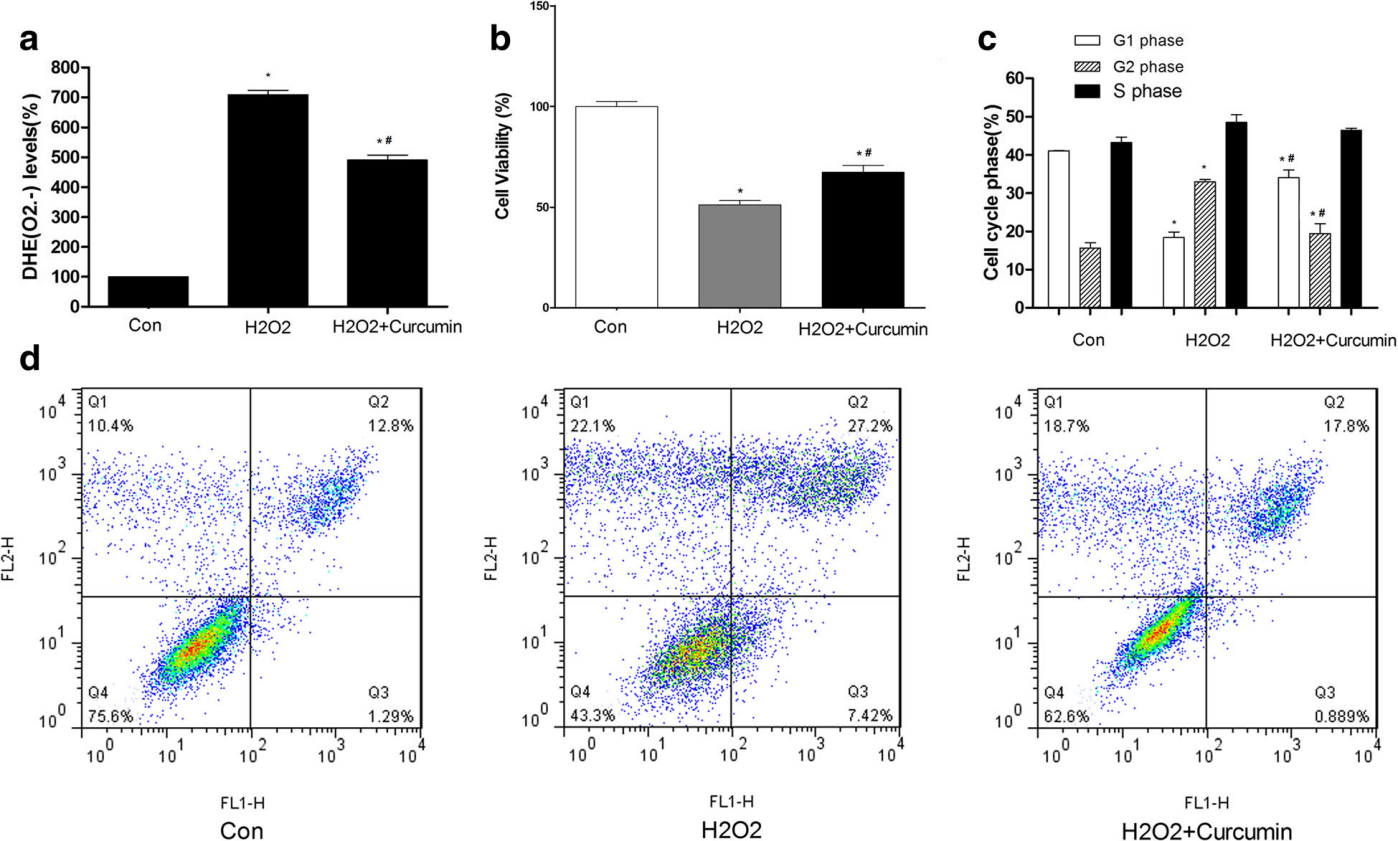
The concentration of intracellular ROS (**a**), cell viability (**b**), cell cycle (**c**), and cell apoptosis (**d**) in the control, H_2_O_2_, and H_2_O_2_ + Curcumin groups. * *P* < 0.05 compared with the control group, # *P* < 0.05 compared with the H_2_O_2_ group

To evaluate the concentration of ROS in each group, the quantified O_2_^−^ level was determined by using fluorescent DHE. As shown in Fig. 2a, the O_2_^−^ level, reflected by red fluorescence, was significantly higher in the model group (*P* < 0.05) compared with the control group, whereas it was much more suppressed in curcumin-treated cells than those in the model group (*P* < 0.05).

### Cell viability and apoptosis analysis

Proliferation was examined in HLEB-3 cells treated with H_2_O_2_, H_2_O_2_ plus curcumin, and untreated HLEB-3 cells. The CCK-8 assay revealed that H_2_O_2_ treatment significantly decreased the viability of HLEB-3 cells (*P* < 0.05), whereas curcumin treatment partly reversed this decrease (*P* < 0.05, Fig. 2b).

The cell cycle analysis (Fig. 2c) indicated that the proportion of HLEB-3 cells in the G1 phase was significantly reduced in the model group and that curcumin could reverse this decrease (*P* < 0.05). Conversely, the proportion of HLEB-3 cells in the G2 phase was remarkably increased in the model group and decreased in the curcumin group (*P* < 0.05). The proportion of cells in the S phase was not significantly different between all three groups (*P* > 0.05).

Flow cytometry was used to determine cellular apoptosis in each group. As shown in Fig. 2d, H_2_O_2_ caused an increase in the number of dead HLEB-3 cells after treatment for 24 h (12.8% vs 27.2%). However, cell apoptosis in the H_2_O_2_ plus curcumin group was decreased (17.8%) compared with that in the H_2_O_2_ group.

### In vitro *mRNA expression of caspase-3, Cox-2, Bax, Bcl-2, c-met, and Slug*

**Fig. 3:**
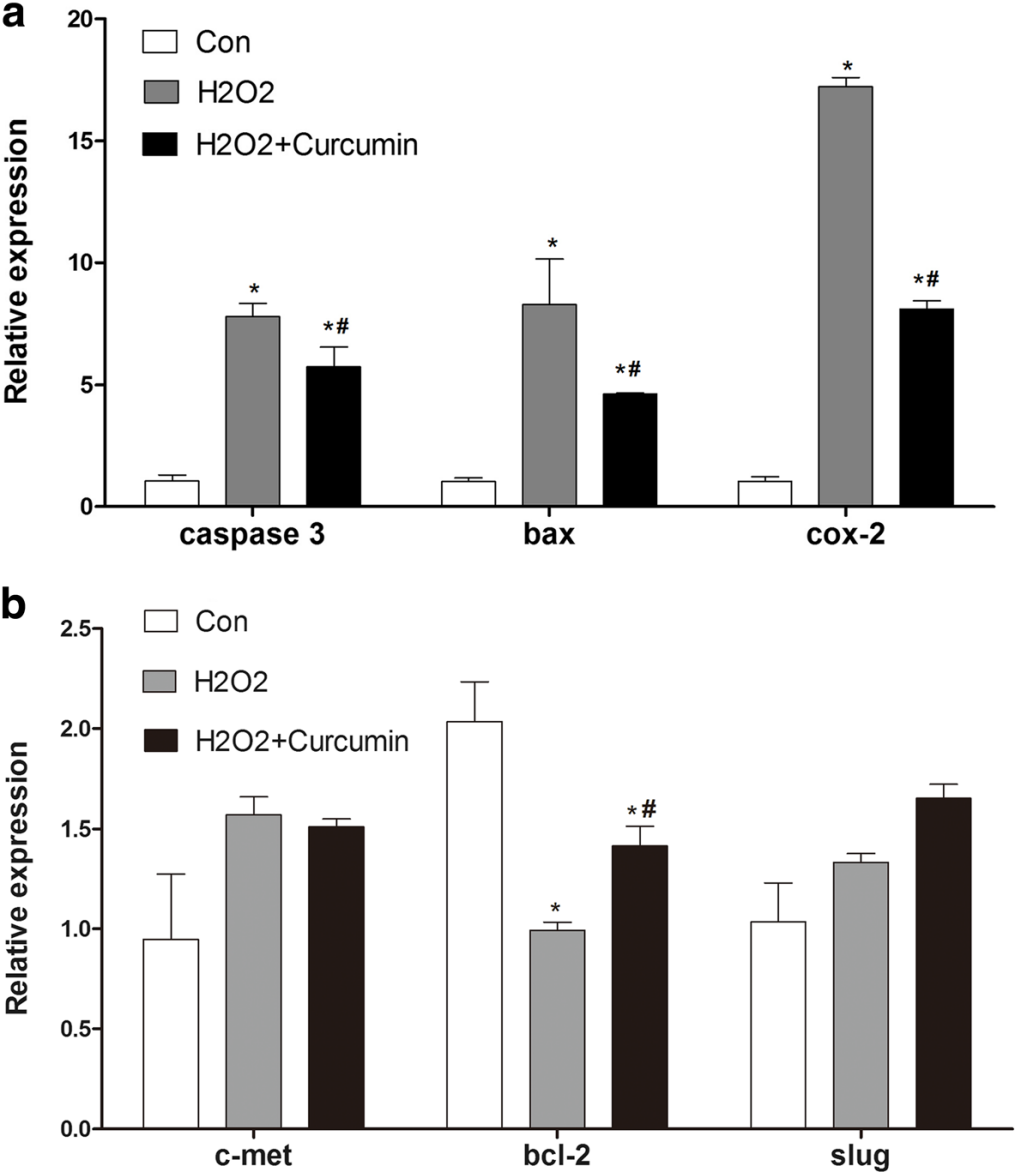
The relative mRNA expression levels of caspase-3, Bax, Cox-2, c-met, Bcl-2 and Slug. * *P* < 0.05 compared with the control group, # *P* < 0.05 compared with the H_2_O_2_ group

As shown in Fig. 3, the relative mRNA expression of caspase-3, Bax, and Cox-2 was significantly increased in the H_2_O_2_ group and the H_2_O_2_ + curcumin group compared with those in the control group (*P* < 0.05). In addition, curcumin treatment significantly decreased the expression levels of these genes compared with the H_2_O_2_ group (*P* < 0.05). The mRNA expression levels of c-met and Slug were increased in the H_2_O_2_ and H_2_O_2_ + curcumin groups, but the changes were not significant (*P* > 0.05).

### Protein expression of MDA, SOD, and CAT

**Fig. 4:**
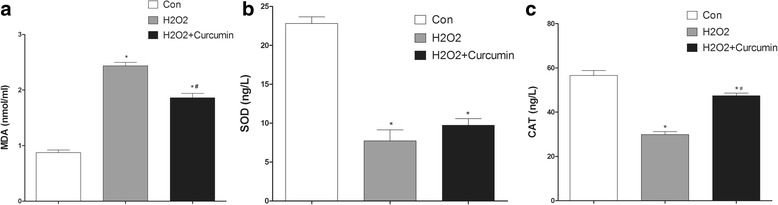
The content of MDA, SOD, and CAT in the control, H_2_O_2_, and H_2_O_2_ + Curcumin groups. * *P* < 0.05 compared with the control group, # *P* < 0.05 compared with the H_2_O_2_ group

The protein expression of MDA, SOD, and CAT in the three treatment groups was determined by using ELISA. As shown in Fig. 4, MDA was significantly upregulated in the H_2_O_2_ group, whereas SOD and CAT were significantly downregulated in the H_2_O_2_ group (*P* < 0.05). In addition, curcumin treatment could reverse the effect of H_2_O_2_ on MDA, SOD, and CAT expression (*P <* 0.05).

## Discussion

Cataract is the leading cause of blindness in the elderly people. Although surgery can be successfully used to remove cataract, the rate of irreversible blindness caused by its complications is highly significant. Previous studies over the past few decades have neglected to screen natural compounds for the potential to ameliorate selenite-induced cataracts. In this study, we investigated the anti-cataractogenic activity of curcumin in in vivo and in vitro studies. The results showed that curcumin could reverse some of the effects of selenite through the regulation of the expression levels of HSP70 and the activities of reactive intermediates, including 8-OHdG, MDA, CAT, SOD, and GSH-Px. In addition, in vitro studies further suggested that curcumin could decrease the concentration of intracellular ROS and attenuate oxidative damage through the regulation of apoptosis-related genes.

The in vivo cataract model was established through the injection of selenium into the eye. This is a classical method for the creation of a cataract model [11, 13] and is used widely in the study of the pathogenesis of senile cataract and the effect of anti-cataract drugs [18]. The results showed that the eye lens isolated from rats injected with selenium took on a white appearance and decreased in transparency, whereas those from untreated rats did not. Interestingly, the administration of curcumin in the cataract model led to a decrease in selenite-induced turbidity in the eye lens. These results were consistent with previous studies and indicated the successful establishment of the cataract models [19].

HSP70, a major member of the Hsp family, is crucial for the maintenance of normal lens microenvironments [20, 21]. It has been widely acknowledged that one of the major triggers factors for cataract formation is the accumulation of excessive free radical generation, which leads to further oxidative stress [22]. These free radicals may cause oxidative damage in the tissue of the anterior eye segment. The expression of HSP70 in the eye lens of rats exposed to selenium was significantly upregulated in our study. This result was consistent with those of Manikandan et al. [23]. Previous studies suggested that HSP70, HSP27, and HSP40 might play a role in the protection of the lens against a variety of stimulants, including oxidative damage, heat shock, and osmotic stress [21, 24, 25]. Interestingly, our results showed that HSP70 expression in the eye lenses of rats exposed to selenium was significantly upregulated and that curcumin could suppress this expression. We hypothesized that this was because HSP70 is a stress-induced protein and curcumin decreases the oxidative stress caused by an accumulation of free radicals; therefore, HSP70 expression was decreased in curcumin group. Correspondingly, the intracellular concentration of ROS was significantly lower in curcumin-treated cells than in H_2_O_2_-treated cells in our study (*P* < 0.05).

In the present study, the activities of CAT, GSH-Px, and SOD were found to be significantly lower in the model group than in the control group in in vivo and in vitro experiments. However, this decrease was partly ameliorated by curcumin. Similarly, low levels of SOD were previously found in diabetic- and selenite-induced cataract models [26, 27], in which low levels of SOD caused irreversible lens damage. These results further supported the antioxidant properties of curcumin. As an end product of lipid peroxidation, MDA is considered to be a toxic compound in the eye owing to its high cross-linking ability with the lipid membrane [12, 28, 29]. Free radicals have the ability to cause lipid peroxidation, which lead to the loss of lens transparency and cataract formation [30]. A previous study demonstrated that thiobarbituric acid reactive substances, which are also the end products of lipid peroxidation, were increased by selenite-induced cataract. As the activity of MDA was decreased in the curcumin group compared with the model group, this study further demonstrated that curcumin could prevent selenite-induced cataractogenesis through a decrease in lipid peroxidation end products.

Reduced glutathione acts as the first line of defence against free radical-mediated damage. As an H_2_O_2_ scavenger, GSH-Px also acts as a membrane barrier for lipid peroxidation in the lens membrane. Studies have shown that glutathione deficiency leads to cataract in experimental animals [31] and our studies have shown that GSH-Px was reduced in the selenium-induced cataract model and increased in the curcumin group.

There are numerous studies that investigate the effect of curcumin on cataract. However, most have focused on the antioxidant effect of curcumin. In this study, we investigated the effect of curcumin on LEC apoptosis. As shown in Fig. 3, the cell viability was decreased in the model group and the percentage of apoptotic cells was increased compared with the control. However, those in the curcumin group were increased and decreased, respectively. Through the analysis of the expression of apoptosis-related genes, we found that the expression levels of caspase-3, Bax, and Cox-2 were significantly upregulated in the model group and the curcumin group compared with those in the control group (*P* < 0.05). However, compared with the model group, the genes were significantly depressed in the curcumin group (*P* < 0.05). Bcl-2, as a repressor of apoptosis, showed the opposite trend.

The caspase cascade and the heterodimerisation of Bcl-2 family proteins are central components of programmed cell death. Bcl-2 family proteins play important roles in the activation of caspases [32]. Bcl-2 and Bax are two major proteins in the Bcl-2 family that repress apoptosis and promote apoptotic functions, respectively. Selenite caused cell apoptosis and when apoptosis occurred, the expression of Bcl-2 was reduced significantly, whereas the expression of Bax was increased in the model group. However, curcumin attenuated the occurrence of apoptosis through the downregulation of Bax and the upregulation of Bcl-2 expression. These data strongly indicated that the Bcl-2 family of proteins may be involved in the process of curcumin protection from ROS-induced oxidative damage.

The epithelial-mesenchymal transition (EMT) is the change in cell phenotype from an epithelial to a fibrocytic morphology. Previous studies suggested anterior LEC underwent EMT-like changes after cataract surgery [33], but these changes might lead to further complications of posterior capsule opacification. The expression of Cox-2 and Slug is a hallmark of the EMT [34]. In our study, the expression of Cox-2 is significantly upregulated in the model group. However, this expression was reduced to a large extent by curcumin. This was somewhat consistent with a previous study that discovered cataractous LEC underwent EMT expression of Cox-2 mRNA and protein, whereas normal LEC did not [35]. As curcumin significantly decreased the expression of Cox-2, we speculated that curcumin might be useful for the prevention of posterior capsule opacification. However, the expression of Slug was not significantly reduced by curcumin. In addition, the expression of c-met was also not significantly different among groups. The molecular mechanism of these changes warranted further investigations.

## Conclusions

This study investigated the cytoprotective nature of curcumin in in vivo and in vitro experiments. The results showed that curcumin attenuated selenite-induced cataract through a reduction in the intracellular production of ROS and the protection of cells from oxidative damage. This study further suggested that curcumin might greatly reduce the occurrence of cataractogenesis as well as prevent posterior capsule opacification.

## Abbreviations

8-OHdG: 8-Hydroxy-2-deoxyguanosine
Bax: Bcl-2 associated X
Bcl-2: B-cell lymphoma 2
CAT: Catalase
CCK-8: Cell counting kit-8
Cox-2: Cyclooxygenase
EMT: Epithelial-mesenchymal transition
FBS: Foetal bovine serum
GSH-Px: Glutathione peroxidase
H_2_O_2_: Hydrogen peroxide
HSP70: Heat shock protein
LEC: Lens epithelial cells
MDA: Malondialdehyde
NO: Nitric oxide
O2^−^: Superoxide anion
OHs: Hydroxyl radicals
ROS: Reactive oxygen species
SOD: Superoxide dismutase
